# Electrophysiological and Behavioral Responses of *Orchestes steppensis* (Coleoptera: Curculionidae) to *Ulmus* Plant Volatiles

**DOI:** 10.3390/plants14010042

**Published:** 2024-12-26

**Authors:** Meng Yang, Qin Li, Guoshuai Zhao, Yalin Liu, Yonggen Lou

**Affiliations:** 1 College of Life Science and Technology, Xinjiang University, Urumqi 830046, China; 15172242757@163.com (M.Y.); 15688998265@163.com (G.Z.); m18749236916@163.com (Y.L.); 2 Xinjiang Key Laboratory of Biological Resources and Genetic Engineering, Urumqi 830046, China; 3 Key Laboratory Biological Crop Pathogens & Insects Zhejiang Pro, State Key Laboratory Rice Biology, Institute Insect Science, Zhejiang University, Hangzhou 310058, China; yglou@zju.edu.cn

**Keywords:** *O. steppensis*, chemical ecology, host preference, volatile compound, GC-EAD, EAG, *Ulmus*

## Abstract

The flea-weevil *Orchestes steppensis* Korotyaev (Coleoptera: Curculionidae) is an Eastern Palaearctic Steppe species, and a serious pest of elm trees (*Ulmus* spp., Ulmaceae) by feeding on the leaves (adults) or mining them heavily (larvae) in Xinjiang, China. In order to search for chemical and ecological pest management practices, the olfactory preferences of *O. steppensis* for leaves of three elm species were investigated. The results revealed that *O. steppensis* has different host preferences for the three elm species: *U. pumila* L. first, followed by *Ulmus laevis* Pall. and *Ulmus densa* Litw. last. Volatile organic compounds from the leaves of the three *Ulmus* species were collected using dynamic headspace adsorption and analyzed through gas chromatography–mass spectrometry (GC-MS). A total of 94 volatile components in the healthy leaves and the infested leaves by *O. steppensis* of the three elm species were identified and analyzed, and 13 active compounds were identified using coupled gas chromatography–electroantennographic (GC-EAD) recording and GC-MS analysis. The response of *O. steppensis* to different concentrations of active compounds was determined using EAG, and the behavioral response to the highest EAG concentration of each active compound was determined. The results demonstrated that 3-hexen-1-ol, 3,7-dimethyl-1,3,6-Octatriene, methyl salicylate, 1-hexanol, and 3-hexen-1-ol, acetate were attractive to *O. steppensis*, while nonanal and 2-hexenal were repellent.

## 1. Introduction

*Ulmus* spp. (Ulmaceae) are drought resistant, cold tolerant, and adaptable, the characteristics that play a very important role in windbreak plantations and sand fixations in desert and semi-desert environments and urban landscapes in Xinjiang (Xinjiang Uyghur Autonomous Region of China) [1,2]. Therefore, they are widely distributed and have also been planted extensively in many urban and rural areas of this region. However, they have been seriously damaged by *Orchestes steppensis* Korotyaev, 2016 (Coleoptera: Curculionidae) in recent years. In 2022, the occurrence area of *O. steppensis* in Hutubi County and Changji City reached 2000 hm^2^ [3]. A large number of pesticides are used to control this pest, but there is little research on chemical ecological control [4]. It is reported that host volatile organic compounds (VOCs) play an important role in monitoring and trapping pest populations, which have been used as chemical and ecological pest management practices for control of Thripidae and Scarabaeidae pests [5,6].

*O. steppensis* is an oligophagous insect with a body length of 2.6~3.2 mm and feeds on the leaves of *Ulmus* spp. (adults) or mines them heavily (larvae), thus making the trees incapable of vigorous growth, and even causing defoliation and death of the lower, small branches [2]. It is widely spread across China, Kazakhstan, Mongolia, North America, and Russia [2,7,8,9]. In China, this pest is distributed in Anhui, Beijing, Gansu, Hebei, Jiangsu, Jilin, Liaoning, Nei Mongol, Ningxia, Shaanxi, Shandong, Shanghai, Tianjin, and Xinjiang [2,10,11]. In Xinjiang, *O. steppensis* completes one generation per year. The overwintering adults emerge from hibernation from late March to early April. After mating, they lay eggs along the main veins of elm leaves. The eggs develop until mid-May when the new generation begins to emerge as adults [12]. The degree of damage of *O. steppensis* to the three elm species is different; the most severe damage occurs to *Ulmus pumila* L., followed by *Ulmus densa* Litw., and finally *Ulmus laevis* Pall. [2].

Insect antennae have tactile and olfactory functions used for searching for host plants and avoiding natural enemies [13]. Herbivorous insects can search for their host for feeding or egg-laying by sensing the VOCs of their host plants [14,15]. GC-EAD, EAG, and olfactory behavioral assays are commonly used to determine the effects of chemicals on the biological activity of insects; GC-EAD and EAG initially screen for active substances and their optimal concentrations, and olfactometers are used to evaluate the biological effects of these active substances and concentrations [16,17].

The aims of our study were to detect whether the host preference of *O. steppensis* to the three elm species was affected by the VOCs of the elm leaves firstly and then to study the VOCs in the healthy leaves and the herbivore-induced plant volatiles (HIPVs) infested by *O. steppensis* to make clear the specific VOCs in the elm leaves attractive or repellent to this weevil pest. Finally, the biological activity of the identified compounds was tested in the indoor bioassay.

## 2. Materials and Methods

### 2.1. Insects and Plants

Adults of *O. steppensis* were collected in the field by net sweeping from April to October 2022~2023 in Urumqi City, Xinjiang, China. Live weevils were placed in an intelligent artificial climate chamber (25 ± 1 °C, 45% ± 5%RH, photoperiod 13L:11D) (BD-PRX-150B; Nanjing Beidi Experimental Instrument Co., Ltd., Nanjing, China). They were fed with fresh *U. pumila* leaves, and the leaves were replaced every 7 d. Three elm (*Ulmus*) species, *U. pumila*, *U. densa*, and *U. laevis*, planted at Xinjiang University were the test plants.

### 2.2. Tested Chemicals

The chemicals used in the experiments are listed in Table 1. The glass materials used during the experiments were heated 1 h at 99.9 °C before and after being used for experimental purposes.

### 2.3. Selection Preferences of the O. steppensis to Leaves of Three Elm Species

A four-arm olfactometer (XLM4-150; Nanjing Xuelai Biotechnology Co., Ltd., Nanjing, China) was used to test the host preference of *O. steppensis* for leaves of three elm species [18,19,20]. Adults were starved for 24 h prior to the experiment, and each weevil adult was tested only once. A female or male adult was chosen at random for the test, and the sexes can be differentiated using morphological characteristics of the genitalia [8,21]. The selection preferences of *O. steppensis* for air (control) and leaves (experimental) of *U. pumila*, *U. laevis*, and *U. densa*, respectively, were first tested. Two upper taste channels of the four-armed olfactometer were closed, and two lower taste channels were open for the test. The experimental channel was connected to 3.0 g fresh, healthy elm leaves as the odor source; the control channel was filled with clean air. Fifty adults of *O. steppensis* per group were tested, and for accuracy, the tests were repeated three times. Secondly, the selection preference of *O. steppensis* of three elm leaves was further tested. Three channels were connected to 3.0 g three elm leaves as the odor source, and one channel was connected to the clean air; 70 adults were tested in each group, and similarly, the tests were conducted in triplicate.

The diameter of the four-armed olfactometer was 150 mm, the length of each odorant channel was 15 cm, and the angle between the two channels was 90°. The gas flow rate of each odorant channel was adjusted to 400 mL/min before the measurement, and after 5 min of aeration, the adult insects were introduced into the insect hole at the top of the four-armed olfactometer and timed when they started to crawl. If the insect crawled through 2/3 of any odorant channel (10 cm from the insect hole) and stayed for more than 30 s or passed through the odorant channel directly within 10 min, it was regarded that the individual chose the substance connected to this odorant channel, and the time when the individual reached the bottle of the odorant source was recorded subsequently; otherwise, it was regarded as unchosen and was not included in the statistical analysis. We found that *O. steppensis* has upward convergence, light convergence, and strong aggregation; therefore, in order to avoid the influence on the experimental results, the following measures need to be taken: firstly, the four-armed olfactometer was placed horizontally, and the activity room and the odorant source bottle were darkened by using a visor, in order to avoid the influence of the light angle and the color of the plant in the bottle on the results of the experiments; secondly, for every 5 insects tested, the four-armed olfactometer needed to be rotated clockwise by 90° to avoid the influence of positional effects; for every 20 insects tested, the testing area was cleaned with anhydrous ethanol and distilled water to avoid the influence of insect source pheromones.

### 2.4. Elm Leaf Volatiles Collection and Analysis

The headspace dynamic technique of Wu et al. (2022) was used to collect elm leaf volatiles with minor modifications [20,22,23]. The microwave oven bags (oven bags, 43 cm × 55 cm, Reynolds, ND, USA) were used to enclose healthy or insect-infested elm branches and sealed. The leaves were not removed from the plants. The original air in the bag was evacuated with an atmospheric sampler, and fresh air was pumped into the bag through an activated carbon tube (5 mm × 8 mm, 200 mm glass tube filled with 1.5 g of 20~40 mesh activated carbon adsorbent) until the bag was full of air. A glass adsorption column with 50 mg of Porapak Q (80–100 mesh) between glass wool plugs at both ends was then attached at the outlet. After 4 h of continuous collection by airflow (1 L/min) through the activated carbon tube, volatiles adsorbed on the Porapak Q adsorbent were eluted with 1 mL of dichloromethane and then 1 mL of n-hexane, respectively. After the elution, it was transferred into a 2 mL Agilent vial and stored in a refrigerator at −20 °C for the use of GC-MS and GC-EAD. The methyl benzoate (100 ng/μL) was used as an internal standard. HIPVs by *O. steppensis* were collected by inoculating 100 *O. steppensis* individuals in the healthy volatiles experiment, allowing them to feed for 24 h, removing the insects, and collecting them again in bags using the method described above. A blank control was set up to prevent the gas components in the bag or in the air from influencing the experiment. The collection of volatiles was repeated three times.

Healthy volatile organic compounds of the three elm species and the HIPVs infested by *O. steppensis* were analyzed using a gas chromatography–mass spectrometer (Agilent, Santa Clara, CA, USA, GC 7890B/MS 5977A). The volatiles were transferred in splitless mode to the analytical column (DB-WAX: 60 m × 0.25 mm ID, 0.25 μm film) placed in the GC oven. The GC oven temperature was initially held at 40 °C for 0.5 min and raised at 10 °C/min to 150 °C and held for 8 min, then 20 °C/min to 240 °C and held for 8 min. At 70 eV, EI-mass spectra were acquired while scanning from *m*/*z* 45 to 650 at a rate of 2.40 scans s^−1^. The MS transfer line and ion source were set to 250 °C and 230 °C, respectively. Identification of healthy VOCs and HIPVs of the three elm species was based on a comparison of mass spectra with those reported in the Agilent MassHunter NIST 14.0 library. Categories of the compounds were determined by referring to [24] and the key functional groups in the compounds. The retention index (RI) of the compounds was calculated by I = 100n + 100 × {[logt’R(X) ‒ logt’R(n)]\[logt’R(n + 1) ‒ logt’R(n)]}. The retention times of normal alkanes of C7-C40 as the standard substance were conducted using the same procedure as the compounds for calculation of the RI of the compounds. Quantitative analysis was performed using the internal standard method to calculate the relative content of each compound. The relative amount of internal standard in each sample could be calculated from the volume of the eluate and its concentration. Each compound in the sample was finally expressed as ng/μL.

Relative content of the tested compound = Chromatographic peak area of the tested compound × Relative content of the internal standard/Chromatographic peak area of the internal standard.

### 2.5. Electrophysiological Responses of O. steppensis Antennae to Elm Leaf Volatiles by GC-EAD

GC-EAD was used to test the electrophysiological responses of *O. steppensis* antennae to elm leaf volatiles [25,26]. The conditions for gas chromatography were the same as those described above for GC-MS. EAG (SYNTECH, Christinenthal, Germany) consists of the stimulus controller CS-55, the MP-15 micromanipulator, the 2-channel programmable signal acquisition controller IDAC-2, the GC/EAD interface temperature controller TC-02, and EC-03 gas phase shunt material heating system. The outlet of the GC column was split at a 1:1 ratio with a universal Y-splitter (Agilent) between the FID and EAD. Healthy *O. steppensis* females and males were taken after 24 h of starvation, and the antennae were removed with a scalpel along the length of about 50 μm from the base and the end of the antennae under the stereomicroscope. The treated antennae were then connected to two saline-filled glass electrodes connected to silver wires at both ends of the tentacle potentiostat. The base of the antenna is connected to the reference electrode, and the end of the antenna is connected to the recording electrode; 2 µL of collected elute of elm leaf volatile compounds were manually injected, and the split method was used for non-split injecting, respectively, using antennae taken from different individual insects.

### 2.6. EAG Responses

The preparation of antennae in the EAG experiment was the same as in the GC-EAD experiment described above. We used standards of compounds that can trigger electrophysiological phenomena in antennae for the GC-EAD experiments. The compounds 2-hexenal, 3,7-dimethyl-1,3,6-octatriene, 3-hexen-1-ol-acetate, 1-hexanol, 3-hexen-1-ol, nonanal, decanoic acid propyl ester, methyl salicylate, 2-methylpropyl ester benzoic acid, guaiacol, benzyl alcohol, phenylethyl alcohol, and 2,4-di-tert-butylphenol were diluted in paraffin liquid to five different concentrations (0.01, 0.1, 1, 10, and 100 mg/mL) for EAG and Y-tube olfactometer behavioral assays. All compounds were obtained from commercial suppliers (Table 1). We did not use all standard compounds because some were not available at the time of our study; 10 μL of the sample were taken to be tested and were dropped evenly on a 1 cm × 3 cm strip of filter paper, and then the solvent was allowed to evaporate for 10 s before it was placed in a glass Pasteur tube as a source of stimulation. The EAG values of the samples were corrected using solvent liquid paraffin as a blank control. The samples were tested in order from low to high concentration. After the baseline had stabilized, the test was started. Each stimulation time was 0.5 s, the stimulation interval was 60 s, and the flow rate of moist air was 1 L/min. Four repetitions were made in the case of males and females of *O. steppensis*, respectively, using antennae taken from different individual insects.

### 2.7. Y-Tube Olfactometer Behavioral Assays

The behavioral responses of *O. steppensis* to the composition of volatile compounds from the host elm leaves were investigated in a Y-tube olfactometer (stem and arms: 15 cm long, respectively, 1 cm internal diam., and 30°Y angle) [25,27]. Behavioral responses of adult *O. steppensis* individuals to 13 GC-EAD experiments with biologically active compounds and concentrations at the highest EAG response values were examined. Filter paper strips dripped with 10 μL of volatile solution were placed at the end of one side of the flavor source, and filter paper strips infused with the same volume of liquid paraffin were placed at the end of the other side of the flavor source as a control. A single healthy adult *O. steppensis*, starved for 24 h, was inserted into the mouth of the main arm tube and left to crawl to start timing. Entering 2/3 of the side arm of the odor source within 5 min and staying for more than 30 s was regarded as selecting the odor source of that side arm, and entering 2/3 of the control side arm and staying for more than 30 s was regarded as selecting the control; if the test bug stayed in the main arm or did not exceed 2/3 of the side wall within 5 min, it was recorded as not selecting for the volatile. Fifty *O. steppensis* were tested for each test solution.

### 2.8. Statistical Analysis

All statistical analyses of data were performed using SPSS 20.0 (IBM). In the selection preferences of the *O. steppensis* to leaves of the three elm species experiment, differences in the number of choices of *O. steppensis* for the same elm leaf and the time to reach the same flavor source bottle between male and female adults of *O. steppensis* were determined by *t*-tests, whereas differences in the number of choices of male and female adults of *O. steppensis* between the experimental groups of *U. pumila*, *U. densa*, and *U. laevis* and the control group were determined by chi-square tests; differences in selection number and selection time of the same sex of *O. steppensis* adults to three elm species and also to the clean air were conducted by one-way ANOVA and Duncan’s multiple comparisons. In the experiment of EAG responses of female and male adults of *O. steppensis* to 13 compounds, differences in the EAG relative values to the same compound at the same concentration between male and female adults of this weevil were determined by *t*-tests, whereas differences in the EAG relative values of male or female adults of *O. steppensis* to different concentration of the same compound were conducted by one-way ANOVA and Duncan’s multiple comparisons. The behavioral response of *O. steppensis* was analyzed using the chi-square test.

## 3. Results

### 3.1. Selection Preferences of O. steppensis for Leaves of Its Three Elm Species Host

**Figure 1:**
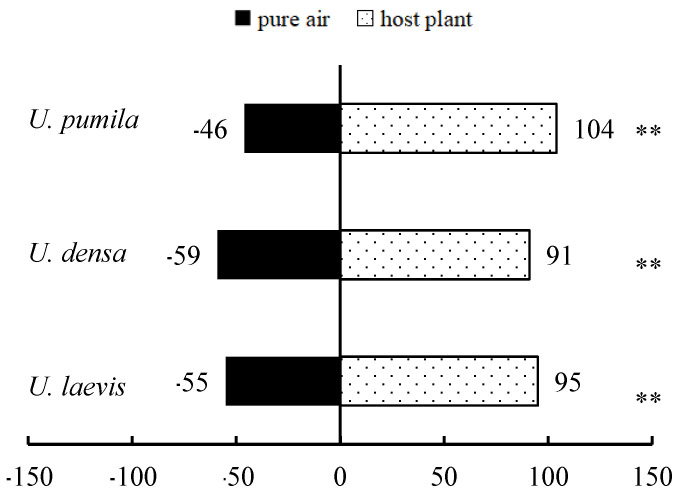
Olfactory responses of *O. steppensis* to three species host plant leaves. Note: ** indicates a significant difference in the level of *p* < 0.01 between the experiment and the control determined by the *x*^2^ test.

The results of the study found that the number of choices of *O. steppensis* for the experimental groups of *U. pumila* (*x*^2^ = 42.32, *p* < 0.01), *U. densa* (*x*^2^ = 23.92, *p* < 0.01), and *U. laevis* (*x*^2^ = 29.04, *p* < 0.01) were all extremely significantly higher than those of the control group (Figure 1), and that the leaves of three species of elm, *U. pumila*, *U. densa*, and *U. laevis*, had an attraction effect on the *O. steppensis*.

There was no significant difference in the reaction time required by adult *O. steppensis* to reach the flavor source between the experimental group and its control group in the three experiments (*p* = 0.52 > 0.05; *p* = 0.42 > 0.05; *p* = 0.75 > 0.05). There was no significant difference (*p* = 0.28 > 0.05; *p* = 0.13 > 0.05; *p* = 0.83 > 0.05; *p* = 0.83 > 0.05; *p* = 0.05 = 0.05; *p* = 0.51 > 0.05) in the time taken by male and female adults to reach the same flavor source bottle (Table 2).

**Figure 2:**
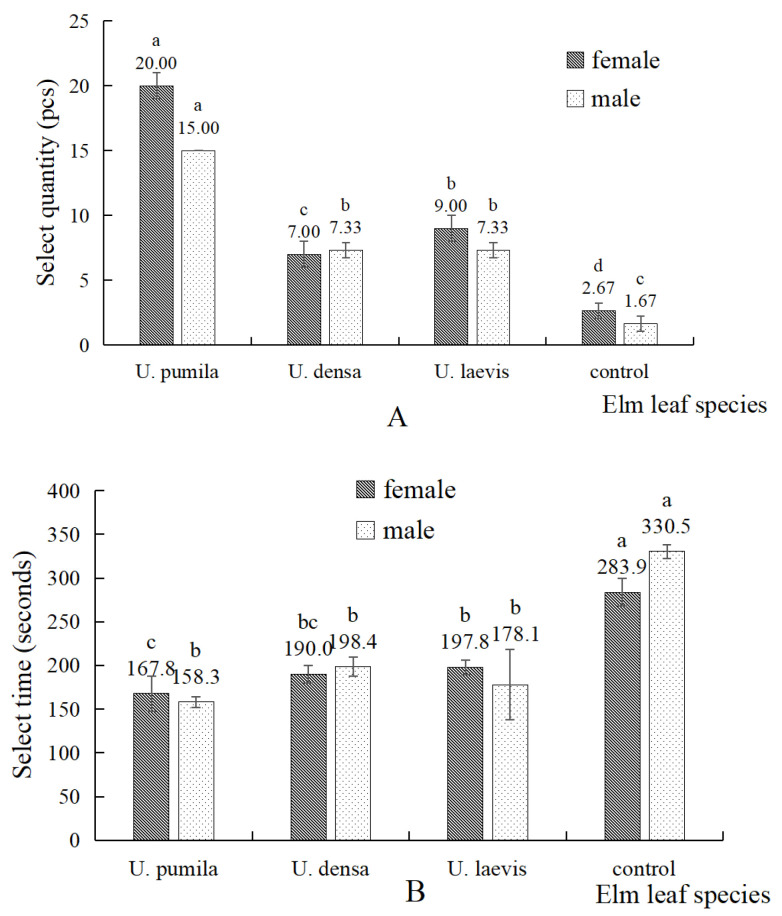
Number of selections of *O. steppensis* adults (**A**) and selection time (**B**) towards the three species of *Ulmus* and the control group. Note: Different small letters indicate that the number or time of selection of different flavor source bottles by the weevil adults of the same sex is significantly different at the *p* < 0.05 level and the difference was determined by one-way ANOVA and Duncan’s multiple comparisons.

The results of the olfactory behavioral responses of *O. steppensis* to three species of elm are shown in Figure 2A, indicating that there were differences in the number of choices made by *O. steppensis* to the leaves of different species. *O. steppensis* females showed the highest number of selections for *U. pumila*, followed by *U. laevis* and finally, *U. densa* (*F* = 196.00, *p* = 0.00 < 0.01); males showed the highest number of selections for *U. pumila* followed by *U. laevis* and *U. densa* (*F* = 359.56, *p* = 0.00 < 0.01). There was no significant difference in the number of selections made by *O. steppensis* males and females for the same flavor source (*p* = 0.35 > 0.05). The results indicate that *O. steppensis* preferences for leaves of different elm species are different, and the preferences are, in descending order, *U. pumila* > *U. laevis* > *U. densa* > blank.

The selection time of *O. steppensis* to three *Ulmus* species is shown in Figure 2B. Among them, *O. steppensis* had the shortest selection time for *U. pumila*, followed by *U. laevis* and finally *U. densa* (*F* = 104.33, *p* = 0.00 < 0.01). The results showed that there was no significant difference between the selection time of male and female *O. steppensis* for the same flavor source (*p* = 0.73 > 0.05).

### 3.2. GC-MS of Elm Leaf Volatiles

A total of 94 compounds were detected by GC-MS in leaf volatiles of three elm species before and after damage by the *O. steppensis*, as shown in Table 3. A total of 10 categories, including 4 benzenes, 21 alcohols, 3 phenols, 4 ethers, 10 aldehydes, 12 acids, 8 terpenes, 3 ketones, 6 alkanes, and 23 ester compounds, were detected. After *U. pumila* was fed on by *O. steppensis*, the percentage of aldehydes increased from 10.36% to 14.67%; the percentage of acids increased from 25.5% to 40.2%; the percentage of terpenes increased from 3.57% to 3.83%; and the percentage of ketones increased from 0.34% to 0.73%. After *U. densa* elm was fed on by *O. steppensis*, the percentage of ether content increased from 0% to 0.47%; the percentage of acid content increased from 31.71% to 38.54%; the percentage of alkane content increased from 4.84% to 5.93%; and the percentage of ester content increased from 13.90% to 15.73%. After *U. laevis* was harmed by *O. steppensis*, the proportion of benzene content increased from 1.64% to 2.51%; the proportion of alcohols and hydrocarbons increased from 12.45% to 21.15%; the proportion of ethers increased from 0.69% to 0.71%; the proportion of aldehydes increased from 1.50% to 12.94%; the proportion of terpenoids increased from 1.38% to 6.97%; the proportion of ketones increased from 0% to 0.36%; and alkane content increased from 1.87% to 3.00%.

The experimental results showed that three elm compound compositions changed significantly in healthy VOCs and HIPVs infested by *O. steppensis* in the leaves of these three elm species. A total of 53 healthy VOCs and 57 HIPVs in the leaves of *U. pumila* were detected. A total of 19 and 25 compounds were detected in *U. densa* leaves before and after feeding by the *O. steppensis*, respectively. Ten new compounds were added to the HIPVs of *O. steppensis* leaves induced by *O. steppensis*; 38 and 54 compounds were detected in *U. laevis* leaves before and after feeding by *O. steppensis*. Twenty-nine new compounds in *U. laevis* were found after feeding by the *O. steppensis*.

**Figure 3:**
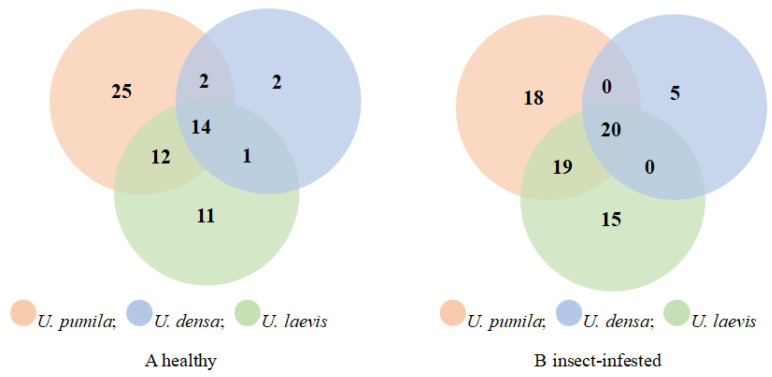
Venn set of VOCs from two leaf states of three elm species. The digits in the figure indicate unique (nonoverlapping color parts) and shared (overlapping color parts) VOC numbers among three elm species.

For the healthy leaves of three elm species, there were 25 compounds specific to *U. pumila*, 2 compounds specific to *U. densa*, and 11 compounds specific to *U. laevis*, as shown in Figure 3A. There were 16 compounds common to *U. pumila* and *U. densa*, 26 compounds common to *U. pumila* and *U. laevis*, and 15 compounds common to *U. densa* and *U. laevis*. The three species of elm shared 14 compounds. In the state of feeding by *O. steppensis* on elm, as shown in Figure 3B, there were 18 compounds specific to *U. pumila*, 5 compounds specific to *U. densa*, and 15 compounds specific to *U. laevis*. There were 20 compounds common to *U. pumila* and *U. densa*, 39 compounds common to *U. pumila* and *U. laevis*, and 20 compounds common to *U. densa* and *U. laevis*. The three species of elm shared 20 compounds.

### 3.3. GC-EAD Analysis

**Figure 4:**
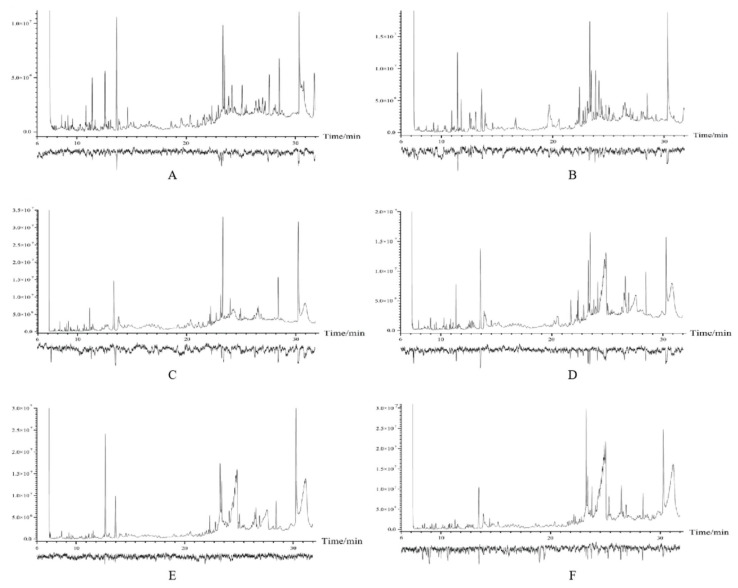
GC-EAD of volatiles from elm leaves by *O. steppensis*. (**A**): Healthy *U. pumila*; (**B**): Infested *U. pumila*; (**C**): Healthy *U. densa*; (**D**): Infested *U. densa*; (**E**): Healthy *U. laevis*; (**F**): Infested *U. laevis*.

GC-EAD analyses with two leaf states of three elm species volatile samples identified volatile compounds that elicited antennal response in *O. steppensis* (Figure 4). The results of the GC-EAD experiments screened a total of 13 effective compounds and their distribution in two leaf states of three elms (Table 4).

The results of the GC-EAD experiment screened a total of 13 effective compounds and their distribution in the two leaf states of the three elms, as shown in Table 4. *U. pumila* healthy and damaged leaves were screened for 13 and 13 effective compounds, respectively; *U. densa* healthy and damaged leaves were screened for 7 and 8 effective compounds, respectively; and *U. laevis* healthy and damaged leaves were screened for 8 and 10 effective compounds, respectively.

### 3.4. EAG Responses of Female and Male Adults of O. steppensis to 13 Single Compounds

**Figure 5:**
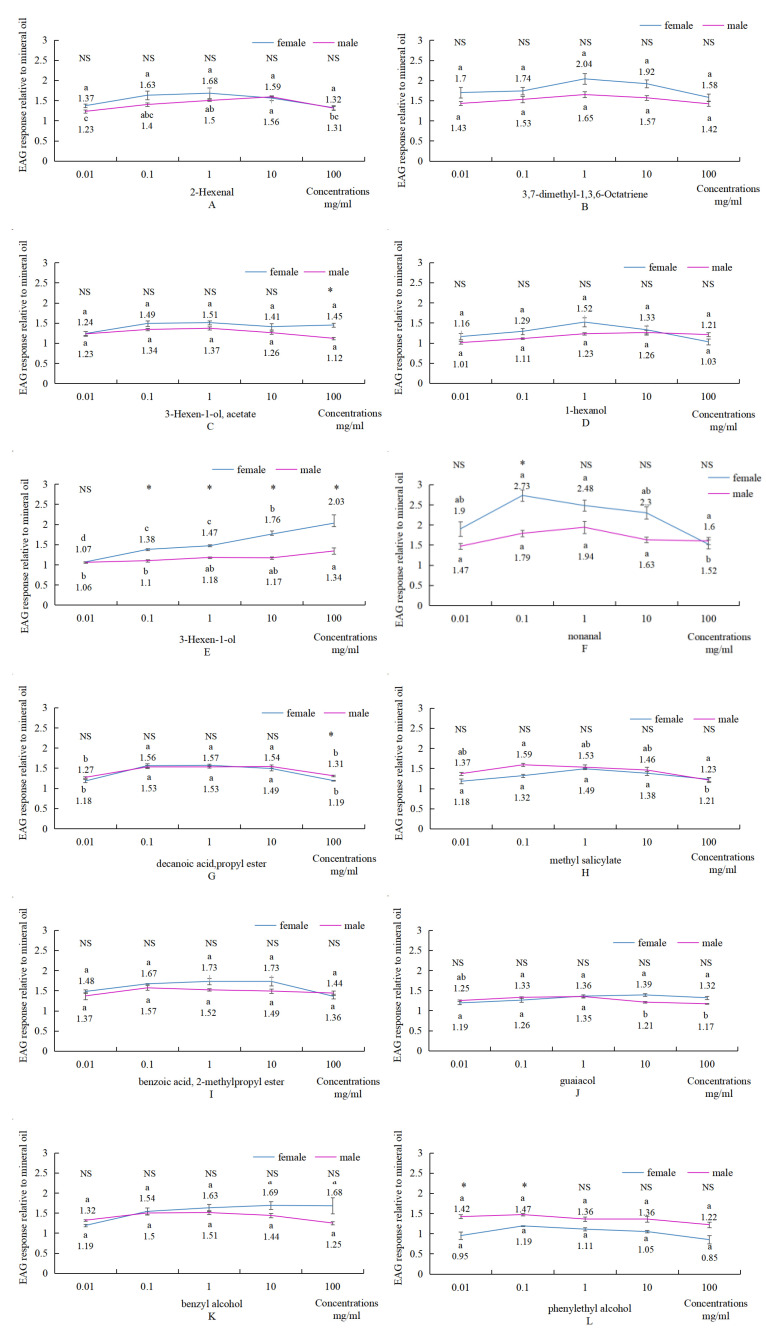

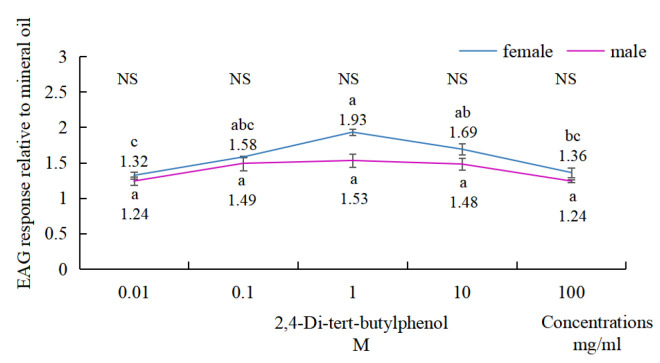
Normalized EAG responses of male and female adults of *O. steppensis* to 13 volatile compounds. (**A**): 2-hexenal; (**B**): 3,7-dimethyl-1,3,6-octatriene; (**C**): 3-hexen-1-ol,acetate; (**D**): 1-hexanol; (**E**): 3-hexen-1-ol; (**F**): nonanal; (**G**): decanoic acid, propyl ester; (**H**): methyl salicylate; (**I**): benzoic acid, 2-methylpropyl ester; (**J**): guaiacol; (**K**): benzyl alcohol; (**L**): phenylethyl alcohol; (**M**): 2,4-di-tert-butylphenol. Notes. Different small letters indicate that EAG responses of female and male adults of *O. steppensis* to different concentrations of the same compound are significantly different at the *p* < 0.05 level, which was determined by one-way ANOVA and Duncan’s multiple comparisons; difference in EAG responses of *O. steppensis* to the same concentrations of the same compound between male and female adults of this weevil pest was determined by *t*-test (NS: *p* > 0.05; *: 0.01 < *p* < 0.05).

The normalized EAG responses of female and male adults of *O. steppensis* to 2-hexenal, 3,7-dimethyl-1,3,6-octatriene, 3-hexen-1-ol, acetate, 1-hexanol, 3-hexen-1-ol, nonanal, decanoic acid, propyl ester, methyl salicylate, Benzoic acid, 2-methylpropyl ester, Guaiacol, Benzyl alcohol, Phenylethyl Alcohol, and 2,4-Di-tert-butylphenol at five concentrations (0.01, 0.1, 1, 10, and 100 mg/mL diluted in paraffin liquid) were measured, as shown in Figure 5.

*O. steppensis* showed EAG responses to all 13 compounds. *O. steppensis* showed the highest EAG relative response values to 1 mg/mL 2-hexenal, 1 mg/mL 1,3,7-dimethyl-3,6-Octatriene, 1 mg/mL 3-hexen-1-ol, acetate, 1 mg/mL 1-hexanol, 100 mg/mL 3-hexen-1-ol, 0.1 mg/mL nonanal, 1 mg/mL decanoic acid, propyl ester, 1 mg/mL methyl salicylate, 1 mg/mL Benzoic acid, 2-methylpropyl ester, 1 mg/mL guaiacol, 1 mg/mL benzyl alcohol, 0.1 mg/mL Phenylethyl Alcohol, and 1 mg/mL 2,4-di-tert-butylphenol had the largest relative EAG response values.

### 3.5. Y-Tube Tests of O. steppensis Choice to Compounds

**Figure 6:**
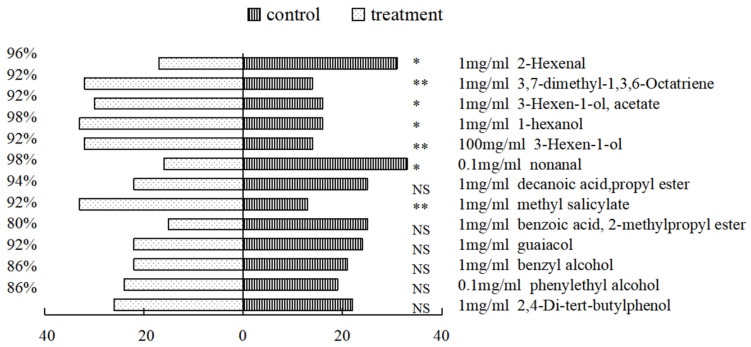
Normalized EAG responses of *O. steppensis* adults to volatile compounds. Note: Y-tube behavior choices of *O. steppensis* towards different compounds at different concentrations determined by the *χ*^2^ test. (NS: *p* > 0.05; *: 0.01 < *p* < 0.05; **: *p* < 0.01).

Different volatile compounds showed significantly different effects on the selection behavior of *O. steppensis*. Behavioral experiments using the optimal concentrations of effective compounds demonstrated that *O. steppensis* prefer to choose 100 mg/mL of 3-hexen-1-ol, 1 mg/mL of 1,3,7-dimethyl-3,6-Octatriene, and 1 mg/mL of methyl salicylate more than in the control group and their selection difference are extremely significant, as shown in Figure 6.

## 4. Discussion

The results of the volatile bioassay showed that when *O. steppensis* searches for host plants, olfactory detection plays a leading role, and the selection rate varies for different host plants. Both male and female *O. steppensis* showed selective preference for *U. pumila*, *U. densa*, and *U. laevis.* The results revealed that *O. steppensis* has different host preferences for the three elm species: *U. pumila* first, followed by *Ulmus laevis* and *Ulmus densa* last. The results were inconsistent with the reports of Zhang (2000) [12] and Li (2018) [2], and they reported that the damage severity in descending order of *O. steppensis* on these three elm species was *U. pumila*, *U. densa*, and *U. laevis*. In this study, we drew a conclusion that the reason why the indoor olfactory selection behavior is not completely consistent with the field damage may be because the current study is only based on the healthy volatiles results of three elm species’ attraction to *O. steppensis*, while the effect of leaf damage on the attraction of *O. steppensis* remains unknown at present. Further investigation is needed to determine whether the behaviors of phytophagous insects–such as feeding, mating, and laying eggs on elm leaves–are influenced by the physical properties (such as size and shape) and chemical composition (such as nutrients) of the leaves [28].

Different tree species have different chemical compositions and relative contents before and after infestation, which constitute different chemical fingerprints of the host plant [29]. In our study, the highest compounds in healthy and post-injury leaves of *U. pumila* were 3-hexen-1-ol and phenylacetaldehyde, and for *U. densa,* HIPVs were benzyl alcohol and 3-hexen-1-ol, respectively, and benzoic acid and phenylacetic acid propyl ester for *U. laevis*. Plant volatiles are mostly mixtures with few individual compounds, and various compounds in certain proportions make up the chemical fingerprints of different plant species. Different plant–insect systems release different volatiles, and the composition of compounds released from the damaged parts of plants is related to their species and phytophagous insect species [30]. At present, there have been some studies on elm volatiles both domestically and internationally, and the results of volatile composition tests differ among different elm species and different parts of the plant. The results of this experiment showed that there were significant differences in the volatiles of the leaves of three elm species. *U. pumila* leaves had the most and richest variety of volatiles before and after damage. Changes in volatiles have an effect on the behavior of phytophagous insects after a plant is damaged [31].

EAG bioassay of volatile compounds showed that adult males and females differed significantly only at partial concentrations of 3-hexen-1-ol, acetate, 3-hexen-1-ol, nonanal, decanoic acid, propyl ester, and phenylethyl alcohol. Different concentrations of the same compound display different physiological activity for herbivorous insects, indicating that the specific concentrations of plant volatiles are important factors that influence insect responses [32]. The antennae potential response of insects only responds to the insect’s sensitivity to volatiles; behavioral experiments are required to determine the specific role of volatiles [33]. In the EAG experiments, the EAG values of females were generally higher than those of males, except for phenylethanol, and the results indicated that *O. steppensis* females were more sensitive to host plant volatiles than males in their antennae potential responses. It has been reported that female insects localize, feed, and lay eggs through host plant volatiles and, therefore, have a greater EAG response to host plant volatiles [34,35]. It is hypothesized that *O. steppensis* females need to search for hosts for oviposition and, therefore, need to be more sensitive to plant volatiles in the environment [36].

Electrophysiological and behavioral results indicated that 100 mg/mL of 3-hexen-1-ol, 1 mg/mL of 3,7-dimethyl-1,3,6-octatriene, methyl salicylate, 1-hexanol, and 3-hexen-1-ol, acetate had an attractive effect on *O. steppensis*; 0.1 mg/mL of nonanal and 1 mg/mL of 2-hexenal had a repellent effect on *O. steppensis*. In previous studies, the results of antennae potentials and behavioral responses were inconsistent, and it was believed that the behavioral selection of insects to volatiles was also influenced by environmental factors, their own physiological conditions, and other aspects [37,38,39]. The electrophysiological and behavioral results of this experiment were also inconsistent, and it was believed that the olfactory localization of *O. steppensis* did not only rely on the antennae sensors but was also affected by other olfactory sensors on the body surface. Although the antennae had an electrophysiological response, there was no associated behavior. Healthy leaf volatiles of *U. pumila*, *U. densa*, and *U. laevis* contained five, two, and three eliciting components, respectively, and the relative content of the two repellent components (2-hexenal and nonenal) in the healthy leaves of *U. pumila* was lower than in that of *U. densa*. There was only one repellent component, nonenal, in the healthy leaves of *U. laevis* compared to the former two elm species. Consequently, *U. pumila* was the most attractive host for *O. steppensis* elm, followed by *U. laevis* and finally *U. densa*. This result is consistent with the results of laboratory experiments on behavioral responses of *O. steppensis* to the leaves of three elm species. It was reported that phytophagous insects rely on a series of compounds in specific ratios for host plant selection [40]. In the current study, the experiment was only conducted on a single component, and the behavioral experiments were not conducted on a mixture of the components. In addition, the test insects were collected in the field, and their age was not clear, which may have interfered with the experimental results to some extent. Whether the optimal concentration of the screened compounds specifically has the effect of trapping *O. steppensis* in the forest needs to be followed up with wind tunnel tests and forest experiments.

## Figures and Tables

**Table 1:** List of the chemicals used in the experiments tested.

No.	Compounds	CAS Number	Purity	Test	Company
1	2-hexenal	6728-26-3	98%	EAG, Y-tube experiments	Macklin ^1^
2	3,7-dimethyl-1,3,6-octatriene	13877-91-3	≥98%	EAG, Y-tube experiments	Macklin ^1^
3	3-hexen-1-ol, acetate	3681-71-8	98%	EAG, Y-tube experiments	Macklin ^1^
4	1-hexanol	111-27-3	99%	EAG, Y-tube experiments	Macklin ^1^
5	3-hexen-1-ol	928-96-1	98%	EAG, Y-tube experiments	Macklin ^1^
6	nonanal	124-19-6	96%	EAG, Y-tube experiments	Macklin ^1^
7	decanoic acid, propyl ester	30673-60-0	98%	EAG, Y-tube experiments	Adamas ^2^
8	methyl salicylate	119-36-8	AR, ≥99%	EAG, Y-tube experiments	Macklin ^1^
9	benzoic acid, 2-methylpropyl ester	120-50-3	99%	EAG, Y-tube experiments	Macklin ^1^
10	guaiacol	90-05-1	GC, >99%	EAG, Y-tube experiments	Macklin ^1^
11	benzyl alcohol	100-51-6	AR, ≥99%	EAG, Y-tube experiments	Macklin ^1^
12	phenylethyl alcohol	60-12-8	AR	EAG, Y-tube experiments	Macklin ^1^
13	2,4-di-tert-butylphenol	96-76-4	97%	EAG, Y-tube experiments	Macklin ^1^
14	paraffin liquid	8012-95-1	AR	EAG, Y-tube experiments	Zhi Yuan ^3^
15	ethanol absolute	64-17-5	AR, 99.7%	EAG, Y-tube experiments	Hu Shi ^4^
16	n-Hexane	110-54-3	HPLC	Collection of plant volatiles	Da Mao ^5^
17	dichloromethane	75-09-2	HPLC	Collection of plant volatiles	Da Mao ^5^
18	methyl benzoate	93-58-3	HPLC	Detection of plant volatiles	Xi Ya ^6^

^1^ Macklin: Shanghai, China; ^2^ Adamas: Shanghai, China; ^3^ Zhi Yuan: Tianjin, China; ^4^ Hu Shi: Shanghai, China; ^5^ Da Mao: Tianjin, China; ^6^ Xi Ya: Shangdong, China.

**Table 2:** The average time for *O. steppensis* to arrive at the flavor bottles of three species host plant leaves: mean ± SEM (s).

	Adult Sex	*U. pumila*	*U. densa*	*U. laevis*
test	female	159.62 ± 15.18	207.35 ± 17.34	215.11 ± 3.86
	male	147.13 ± 11.95	194.89 ± 15.71	177.07 ± 11.00
control	female	144.04 ± 10.33	187.24 ± 9.54	203.51 ± 7.93
	male	184.61 ± 23.79	203.33 ± 25.74	182.04 ± 19.33

**Table 3:** Identified volatile components released from two leaf states of three elm species and their Quantities (ng/μL) relative to the internal standards: mean ± SEM.

No.	Retention Time (min)	Retention Index (RI)	Compound	Categories	*U. pumila*		*U. densa*		*U. laevis*	
					Healthy	Insect-Infested	Healthy	Insect-Infested	Healthy	Insect-Infested
1	7.595	974	glyceric acid	Acid	-	-	-	0.43 ± 0.04 a*	0.03 ± 0 A*	-
2	7.976	1000	glycolaldehyde dimethyl acetal	Aldehyde	-	-	-	-	-	0.63 ± 0.05 a*
3	8.202	1014	toluene	Benzene	0.29 ± 0.06 A	0.33 ± 0.02 b	-	-	-	0.68 ± 0.12 a*
4	8.547	1036	3-methylcyclopentanol	Alcohol	0.2 ± 0.06 A	0.13 ± 0.01 b	-	-	0.07 ± 0.01 A	0.68 ± 0.04 a*
5	9.722	1105	p-xylene	Benzene	0.08 ± 0.01 A	0.35 ± 0.06 a*	-	-	-	0.27 ± 0.04 a*
6	9.903	1116	bicyclo[2.1.1]hexan-2-ol, 2-ethenyl-	Alcohol	0.21 ± 0.06 A	0.3 ± 0.02 c	-	1.86 ± 0.21 a*	0.02 ± 0 B	0.97 ± 0.14 b*
7	9.948	1119	trans-1(7),8-p-menthadien-2-ol	Alcohol	-	-	-	-	0.02 ± 0 A*	-
8	10.432	1147	heptanal	Aldehyde	0.7 ± 0.06 A	1.33 ± 0.24 a	0.75 ± 0.1 A	1.65 ± 0.01 a*	-	1.22 ± 0.09 a*
9	10.528	1152	cyclopentanone, 2-(1-methylpropyl)-	Ketone	0.16 ± 0.02 A	0.27 ± 0.1 a	-	-	-	-
10	10.722	1163	2-hexanol	Alcohol	0.21 ± 0 A	0.28 ± 0.04 b	-	-	-	1.21 ± 0.04 a*
11	10.727	1164	(+)-dipentene	Terpenoid	-	-	-	-	0.18 ± 0.01 A*	-
12	11.003	1179	2-hexenal	Aldehyde	1.37 ± 0.18 A	4.36 ± 0.76 a*	2.12 ± 0.2 A	5.94 ± 0.21 a*	-	1.77 ± 0.02 b*
13	11.172	1188	3,7,11-trimethyl-1-dodecanol	Alcohol	0.14 ± 0.04 A	0.19 ± 0.01 b	-	-	-	1.57 ± 0.02 a*
14	11.261	1193	1,3,6-octatriene, 3,7-dimethyl-	Terpenoid	0.25 ± 0.07 A	1.77 ± 0.02 a*	-	-	-	-
15	11.776	1223	tetradecane	Alkane	0.17 ± 0.03 A*	-	-	-	-	-
16	12.075	1241	2,3,5,8-tetramethyldecane	Alkane	0.19 ± 0.04 A*	-	-	-	-	-
17	12.079	1241	vanillin	Aldehyde	-	-	-	-	-	1.21 ± 0.11 a*
18	12.105	1242	cis-2-penten-1-ol	Alcohols	-	1.57 ± 0.02 a*	-	-	-	-
19	12.153	1245	3-hexen-1-ol, acetate(z)	Esters	1.52 ± 0.16 A*	1.06 ± 0.02 a	-	-	3.25 ± 0.75 A*	-
20	12.461	1263	(z)-hex-2-enyl acetate	Esters	0.26 ± 0.05 A	0.49 ± 0.04 a*	-	-	-	-
21	12.673	1275	1-hexanol	Alcohols	0.4 ± 0.06 A	1.99 ± 0.11 a*	-	-	-	-
22	13.217	1305	3-hexen-1-ol, (z)-	Alcohols	4.49 ± 0.54 B	4.31 ± 0.31 c	10.87 ± 1.09 A	23.93 ± 0.62 a*	2.56 ± 0.56 B	16.82 ± 0.36 b*
23	13.413	1317	nonanal	Aldehyde	0.39 ± 0.11 B	1.88 ± 0.37 c*	6.17 ± 1.15 A	13.38 ± 0.54 a*	0.31 ± 0.05 B	7.9 ± 0.95 b*
24	13.650	1331	citronellol	Terpenoid	-	-	-	-	0.3 ± 0.08 A	6.63 ± 0.49 a*
25	14.205	1363	2,6,10-trimethyldodecane	Alkane	0.69 ± 0.04 A*	0.38 ± 0.04 b	-	-	0.26 ± 0.04 B	1.91 ± 0.44 a*
26	14.282	1368	2,6,10-trimethyltridecane	Alkane	-	-	-	-	-	2.27 ± 0.14 a*
27	14.546	1382	trans-2-ethyl-2-hexen-1-ol	Alcohol	-	0.25 ± 0.04 a*	-	-	-	-
28	14.733	1393	1,2-15,16-diepoxyhexadecane	Ether	-	-	-	-	-	0.38 ± 0.05 a*
29	14.763	1394	2,3-dimethylcyclohexanol	Alcohol	0.28 ± 0.07 A*	-	-	-	-	-
30	15.840	1452	linalool	Terpenoid	-	0.2 ± 0.01 a*	-	-	-	-
31	16.171	1469	benzaldehyde	Aldehyde	0.62 ± 0.04 A	2.09 ± 0.1 a*	-	-	-	2.31 ± 0.2 a*
32	18.162	1558	geranyl isovalerate	Ester	0.73 ± 0.06 A*	0.3 ± 0.05 b	-	-	-	2.74 ± 0.44 a*
33	18.554	1574	nonanoic acid, propyl ester	Ester	0.57 ± 0.11 A	0.64 ± 0.01 b	-	2.01 ± 0.33 a*	0.38 ± 0.08 A	2.61 ± 0.01 a*
34	19.081	1595	benzeneacetaldehyde	Aldehyde	1.28 ± 0.07 B	7.45 ± 0.54 a*	2.74 ± 0.06 A*	0.44 ± 0.13 c	0.21 ± 0.01 C	2.08 ± 0.23 b*
35	19.353	1605	(e)-2-hexenyl benzoate	Ester	0.41 ± 0.03 A	-	-	-	0.08 ± 0.01 B	1.62 ± 0.09 a*
36	19.714	1617	benzoicacid, 1-methylethylester	Ester	-	-	-	3.27 ± 0.47 a*	-	-
37	19.887	1623	benzoic acid, ethyl ester	Ester	0.96 ± 0.1 B	2.29 ± 0.19 b*	3.69 ± 0.59 A	7 ± 0.87 a*	0.85 ± 0.03 B	3.73 ± 0.19 b*
38	20.068	1628	radicinin	Ketone	-	-	-	-	-	0.31 ± 0.01 a*
39	20.521	1643	patchouli alcohol	Terpenoid	-	0.42 ± 0.04 a*	-	-	-	-
40	21.060	1660	tridecanedial	Aldehyde	0.54 ± 0.04 A*	-	-	-	-	-
41	21.080	1660	palmitic acid	Acid	-	-	-	-	0.21 ± 0.01 A*	-
42	21.160	1663	decanoic acid propyl ester	Ester	0.62 ± 0.05 A*	0.46 ± 0.02 c	-	1.17 ± 0.11 b*	0.24 ± 0.05 B	1.78 ± 0.14 a*
43	21.212	1664	7-epi-cis-sesquisabinene hydrate	Alcohol	-	-	-	-	-	1.38 ± 0.32 a*
44	21.392	1670	β-pinene	Terpenoid	-	0.28 ± 0.07 a*	-	-	-	-
45	21.460	1672	e-3-pentadecen-2-ol	Alcohol	-	-	-	-	-	2.5 ± 0.79 a*
46	22.404	1700	methyl salicylate	Ester	0.38 ± 0.14 AB	0.41 ± 0.07 c	0.67 ± 0.05 A	1.59 ± 0.07 b*	0.1 ± 0.01 C	3.25 ± 0.52 a*
47	22.542	1703	benzoicacid,2-methylpropylester	Ester	0.55 ± 0.07 B	2 ± 0.03 b	1.92 ± 0.08 A	4.03 ± 0.24 a	0.52 ± 0.03 B	2.17 ± 0.02 b*
48	22.615	1705	4-ethyl-4-methyl-2-pentadecyl-1,3-dioxolane	Ether	-	-	-	-	-	1.38 ± 0.11 a*
49	22.620	1705	2-pentadecyl-1,3-dioxocane	Ether	0.2 ± 0.05 A*	-	-	0.9 ± 0.33 a*	0.24 ± 0.01 A*	-
50	22.957	1714	o-toluylic acid, 2-butyl ester	Esters	0.37 ± 0.04 A	0.84 ± 0.04 a*	-	-	0.16 ± 0.01 B	1.35 ± 0.23 a*
51	23.117	1718	dipropyl succinate	Esters	0.56 ± 0.05 B	1.08 ± 0.03 b*	1.67 ± 0.16 A*	-	0.41 ± 0.06 B	1.66 ± 0.1 a*
52	23.402	1725	guaiacol	Phenol	0.31 ± 0.16 A	1.53 ± 0.2 a*	-	-	-	1.72 ± 0.28 a*
53	23.485	1727	benzoic acid, 4-methyl-, propyl ester	Ester	-	1.12 ± 0.28 a*	-	-	-	-
54	23.550	1728	phenylacetic acid propyl ester	Ester	3.01 ± 0.35 A	7.03 ± 0.73 b*	4.7 ± 0.66 A	9.34 ± 0.73 b*	3.36 ± 0.55 A	42.54 ± 3.37 a*
55	23.610	1730	m-toluylic acid, 2-butyl ester	Ester	0.24 ± 0.08 B	0.84 ± 0.24 a	-	1.89 ± 0.29 a*	0.55 ± 0.06 A	4.38 ± 1.8 a
56	23.687	1732	benzyl alcohol	Alcohol	2.12 ± 0.32 B	2.71 ± 0.18 b	13.47 ± 1.18 A	11.38 ± 0.59 a	1.66 ± 0.26 B	11.6 ± 2.06 a*
57	23.774	1734	pentadecanal	Aldehyde	-	-	-	-	-	0.19 ± 0.05 a*
58	23.847	1736	3-methylene-1-oxa-spiro[4.5]decan-2-one	Ketone	-	0.58 ± 0.02 a*	-	-	-	0.59 ± 0.21 a*
59	24.056	1741	phenylethyl alcohol	Alcohol	0.65 ± 0.06 A	2.59 ± 0.47 b*	-	-	-	8.91 ± 0.46 a*
60	24.370	1748	4-phenyltoluene	Benzene	1.24 ± 0.08 B	2.73 ± 0.17 b*	3.21 ± 0.32 A	5.25 ± 0.1 a*	0.57 ± 0.04 C	5.25 ± 0.86 a*
61	24.545	1752	2-hexadecanol	Alcohol	0.32 ± 0.02 A	1.47 ± 0.05 b*	-	3.11 ± 0.32 a*	-	1.78 ± 0.36 b*
62	24.598	1754	myristic acid	Acid	-	-	-	-	0.48 ± 0.05 A*	-
63	24.644	1755	17-octadecynoic acid	Acid	-	0.63 ± 0.13 a*	-	-	-	-
64	24.654	1755	13-tetradecynoic acid methyl ester	Ester	0.19 ± 0.01 A*	-	-	-	-	-
65	24.909	1761	fumaric acid, ethyl 2-methylallyl ester	Ester	-	-	-	-	-	4.54 ± 0.3 a*
66	24.995	1763	octadecanoic acid	Acid	-	-	-	7.5 ± 1.22 a*	-	-
67	25.233	1768	methyl myristate	Ester	0.36 ± 0.02 A	1.04 ± 0.07 b*	-	-	-	2.77 ± 0.24 a*
68	25.290	1770	2-pentyl-3-methyl-2-cyclopenten-1-one	Terpenoid	0.92 ± 0.07 A	1.02 ± 0.08 a	-	-	-	1.85 ± 0.36 a*
69	25.540	1775	1-hexadecanol	Alcohol	0.27 ± 0.07 A	0.62 ± 0.15 a	-	-	-	-
70	25.576	1776	2-pentadecanone, 6,10,14-trimethyl-	Terpenoid	0.52 ± 0.05 A	0.78 ± 0.08 b	-	-	-	8.72 ± 0.72 a*
71	25.654	1778	13-heptadecyn-1-ol	Alcohol	-	-	-	-	-	1.33 ± 0.41 a*
72	25.660	1778	elaidic acid	Acid	-	-	-	-	0.24 ± 0.03 A*	-
73	25.763	1780	trans-13-octadecenoic acid	Acid	-	-	-	-	0.45 ± 0.01 A*	-
74	26.189	1790	7-hexadecyn-1-ol	Alcohol	-	0.19 ± 0.01 a*	-	-	-	-
75	26.551	1798	biphenyl	Benzene	-	-	0.7 ± 0.06 A*	-	-	-
76	26.693	1802	5-octadecenal	Aldehyde	-	-	-	-	-	14.61 ± 3.7 a*
77	26.713	1802	cis-10-heptadecenoic acid	Acid	-	-	-	-	1.16 ± 0.14 A*	-
78	26.819	1807	cis-3-hexenyl benzoate	Ester	-	-	-	-	0.76 ± 0.15 A*	-
79	26.887	1809	1-hexadecanol,2-methyl-	Alcohol	-	0.19 ± 0.01 b*	-	0.9 ± 0.07 b*	-	3.41 ± 1.18 a*
80	26.909	1810	9,12,15-octadecatrienoic acid, (z,z,z)-	Acid	-	-	3.38 ± 0.49 A*	-	-	-
81	27.148	1820	oleic acid	Acid	0.56 ± 0.04 A	0.94 ± 0.07 b*	-	-	0.57 ± 0.11 A	5.12 ± 0.04 a*
82	27.280	1825	2-methyl-4-(2,6,6-trimethylcyclohex-1-enyl)but-2-en-1-ol	Alcohol	-	0.18 ± 0.02 a*	-	-	-	-
83	27.755	1844	hexadecanoic acid, methyl ester	Ester	1.22 ± 0.17 A*	0.34 ± 0.03 b	0.64 ± 0.09 B*	-	1.13 ± 0.15 A*	0.5 ± 0.01 a
84	28.121	1858	2-methoxy-5-[(e)-1-propenyl]phenol	Phenol	0.22 ± 0.03 A	0.42 ± 0.02 a*	-	-	-	-
85	28.164	1860	(z)-9-hexadecenoic acid methyl ester	Ester	-	-	-	-	0.05 ± 0.01 A*	-
86	28.299	1865	2,6,10-trimethyltetradecane	Alkane	0.47 ± 0.08 A*	-	-	-	0.21 ± 0.06 A	2.53 ± 0.52 a*
87	28.480	1872	3-ethoxy-3,7-dimethyl-1,6-octadiene	Ether	-	0.18 ± 0.02 a*	-	-	-	-
88	28.650	1879	2,4-di-tert-butylphenol	Phenol	1.89 ± 0.05 B	3.04 ± 1.06 b	7.33 ± 0.17 A	7.97 ± 0.51 a	1.43 ± 0.33 B	8.24 ± 0.08 a*
89	28.895	1888	methyl 14-methylhexadecanoate	Ester	0.37 ± 0.06 A*	-	-	-	-	-
90	29.046	1893	1,2-propnediol,3-(tetradecyloxy)	Alcohol	-	0.43 ± 0.07 a*	-	-	-	-
91	30.016	1946	linoleic acid	Acid	-	-	1.04 ± 0.01 A	3.44 ± 0.4 a*	1.07 ± 0.03 A*	-
92	30.296	1962	methyl stearate	Ester	0.11 ± 0.01 A	0.15 ± 0.01 b	-	-	-	1.43 ± 0.1 a*
93	30.486	1973	benzoic acid	Acid	11.5 ± 4.37 B	45.32 ± 7.19 b*	25.89 ± 2.78 A	62.88 ± 3.65 a*	10.51 ± 4.51 B	36.97 ± 2.95 b*
94	30.961	2000	n-eicosane	Alkane	1.02 ± 0.25 B	0.88 ± 0.05 b	4.63 ± 0.4 A	11.42 ± 2.46 a	0.18 ± 0.02 B	0.69 ± 0.17 b*

Note: “-” indicates that the compound was not detected. Different capital letters indicate significant differences in the volatile content of three healthy leaves. Lower case letters indicate significant differences in the volatile content of three leaves damaged by *O. steppensis*, and * indicates significant differences in the volatile content of leaves before and after damage to the same species of elm.

**Table 4:** Distribution of 13 active compounds in the volatiles of two leaf states of three elms by GC-EAD experiments.

No.	Retention Time (min)	Compound	*U. pumila*		*U. densa*		*U. laevis*	
			Healthy	Insect-Infested	Healthy	Insect-Infested	Healthy	Insect-Infested
1	11.003	2-hexenal	+	+	+	+	+	+
2	11.261	3,7-dimethyl-1,3,6-octatriene	+	+	-	-	-	-
3	12.153	3-hexen-1-ol,acetate	+	+	-	-	-	-
4	12.673	1-hexanol	+	+	-	-	-	-
5	13.217	3-hexen-1-ol	+	+	+	+	+	+
6	13.413	nonanal	+	+	+	+	+	+
7	21.16	decanoic acid, propyl ester	+	+	-	+	+	+
8	22.404	methyl salicylate	+	+	+	+	+	+
9	22.542	benzoic acid, 2-methylpropyl ester	+	+	+	+	+	+
10	23.402	guaiacol	+	+	-	-	-	+
11	23.687	benzyl alcohol	+	+	+	+	+	+
12	24.056	phenylethyl alcohol	+	+	-	-	-	+
13	28.65	2,4-di-tert-butylphenol	+	+	+	+	+	+

Note: “+” in the table means that an antennae response was detected, and “-” means that no antennae response was detected.

## Data Availability

Data is contained within the article.
